# Editorial: Historical Roots of Psychopathology

**DOI:** 10.3389/fpsyg.2016.00905

**Published:** 2016-06-14

**Authors:** Diogo Telles-Correia, Daniel Sampaio

**Affiliations:** Faculty of Medicine, University of LisbonLisbon, Portugal

**Keywords:** psychopathology, history of psychopathology, philosophy of psychiatry, symptoms, mental disorders

Mental illness and mental symptoms depend on a construction that results from the decisions of certain social agents, which, in a specific social and historical context, according to an epistemological framework (how symptoms and disorders are constructed and detected) and an ontological framework (how they are defined, what they consist of), identify the behaviors which make up a symptom or a disorder (Berrios, 2011). Only after these theoretical hypotheses of mental symptom and disorder have been outlined are the data which empirically validate them searched and found. Therefore, the objects of psychiatry (mental symptom and disorder) being the result of a social conjecture and a philosophical perspective rooted in a specific time, they should also be studied with elements from social and human sciences (history, sociology, philosophy) (Telles-Correia, 2015).

Besides these elements, clinical experience is essential to find out new forms of presentation, as well as to name new clinical behaviors and manifestations. After all, this was the fundamental method of the great psychopathologists and nosologists of the late Nineteenth century and early Twentieth century, such as Falret, Kalbaum, Kraeplin, etc. (Jaspers, 1963; Goas, 1966).

These theoretical hypotheses, born out of clinical experience together with the historical, sociological and philosophical analysis of previously established theories are connected to reality through empirical validation. The latter aims to show that the said symptoms/diagnoses do really exist (Zachar, 2012).

According to Zachar (2012), there are two major paradigms of empirical validation. The first paradigm is the medical model. According to this model, validation includes the study of the natural history of disorders (which show to be consistent in terms of natural history with the proposed diagnosis), studies of family aggregation (which show a greater influence of heredity in the proposed diagnosis) and the search for neurobiological causality. This model was the basis for the development of classification systems such as the DSM.

At the same time a psychological model was developed. In this psychological/psychometric model current validation processes follow a different paradigm. According to this model, psychological/psychiatric variables are latent variables (which cannot be measured/observed directly but need instead to be assessed through other component variables). The validation of the instruments which measure this latent variable includes complex statistical methods (in the search for construct validity and criterion validity).

These two kinds of validation are not exclusive and nowadays there is a tendency for them to coexist and combine.

In recent years, the search for empirical validation has reached proportions never seen before, generally in line with the structures of diagnosis universally accepted. The so called content validation was thus devaluated. In this approach, the theoretical hypotheses are reassessed based on an important revision of the history and evolution of the established concepts (which comprises the sociological movements and the ontological and epistemological philosophical frameworks of the different authors) and also on the new data from updated clinical practice. With the devaluation of content validation, the current guidelines are considered to be beyond criticism and irrefutable. Most of the times the technicians involved in the validation of psychometric instruments aimed at evaluating a specific concept which has been established decades ago (sometimes more than a 100 years ago) do not know the origin of these concepts nor the history of their development thoroughly (nor if they are socially, historically and philosophically appropriate nowadays). The same applies to the researchers who try to find the anatomical/chemical correlates of psychiatric manifestations but often do not grasp the conceptual basis of the object studied nor the validity of the methods used to detect the said object, nor have they in their team a member able to explain this to them (Telles-Correia, 2015).

New advances of the neuroscience supported by a refined, reliable and valid phenotyping (e.g., at the level of symptoms and not at the level of disorders), are bringing some promising results. The mapping of clinical phenomenology on specific brain dysfunction is now becoming plausible and the resulting functional psychopathology may in the future significantly replace the present nosology (Jablensky, 2010).

Nevertheless, as Andreasen (2007) points out: “Applying technology without companionship of wise clinicians with specific expertise in psychopathology will be a lonely, sterile and perhaps fruitless enterprise.”

Some of the chapters of this Ebook deal with aspects which are essential to the historical understanding of mental symptoms and disorders.

The first text of this topic will briefly review the fascinating history of Asperger syndrome: why it was born, its tumultuous existence, and its downfall.

The second text presents an historical overview of the understanding of Obsessive Compulsive Disorder, highlighting the advances in neuroscience and how they influenced current perspectives on the nosology of this disorder.

The third text reviews many historical sources about the understanding and treatment of mental disorders in the early modern England and connects these with current trends in mental health care.

The fourth text will show a critical review about psychopathology classification systems on Sexual orientation and gender identity and argues for the broader respect and value of the diversity of human sexuality and of gender expressions.

The fifth text will review the history of histrionic personality disorder, one of the most ambiguous diagnostic categories in psychiatry reflecting attitudes about health, religion and gender across time.

The sixth text aims to review the evolution of the term “hallucination” up to present time, highlighting the difficulty in both defining and limiting this concept ever since its first appearance.

The seventh text presents some elements of the Freudian thinking on psychosis. Can the psychotic individual be invaded by a pulsating unconscious which demands a symbolic mediation?

The eighth text reflects recent changes in the Brazilian public policies for mental health since Diagnostic and Statistic Manual of Mental Disorders was introduced which might disregard the subject and its personal history.

The ninth text aims at reviewing the contributions by the different authors to the construction of the term “melancholia,” throughout history, where it has been associated not only to affective disorders but also to abnormal beliefs.

## Author contributions

DT: literature review and elaboration of the final paper. DS: literature review.

### Conflict of interest statement

The authors declare that the research was conducted in the absence of any commercial or financial relationships that could be construed as a potential conflict of interest.
